# Microbiological Sampling in Total Knee Arthroplasty After Post-Traumatic Osteoarthritis: Rate of Periprosthetic Joint Infection and the Debate Around Sampling Unremarkable Tissue

**DOI:** 10.3390/microorganisms13071690

**Published:** 2025-07-18

**Authors:** Felix Erne, Leonard Grünwald, Tina Histing, Philipp Hemmann

**Affiliations:** 1 Siegfried Weller Research Institute, BG Unfallklinik Tuebingen, Department of Trauma and Reconstructive Surgery, Eberhard Karls University Tuebingen, Schnarrenbergstr. 95, 72076 Tuebingen, Germany; 2 Department of Orthopedics and Traumatology, Diakonie-Klinikum Hospital, Rosenbergstr. 38, 70176 Stuttgart, Germany; 3 BG Unfallklinik Tuebingen, Department of Trauma and Reconstructive Surgery, Eberhard Karls University Tuebingen, Schnarrenbergstr. 95, 72076 Tuebingen, Germany; 4 Department of Joint Replacement, General and Rheumatic Orthopaedics, Orthopaedic Clinic Markgroeningen gGmbH, Kurt-Lindemann-Weg 10, 71706 Markgroeningen, Germany

**Keywords:** traumatology, osteoarthritis, knee, total knee arthroplasty, periprosthetic joint infection, biofilm, device-related infections, prosthesis, joint infections, implants

## Abstract

Background: Proximal tibial fractures can lead to post-traumatic osteoarthritis (PTOA), and subsequent total knee arthroplasty (TKA) in such patients is associated with elevated complication rates. A two-stage approach, involving the elective removal of osteosynthetic hardware prior to TKA, is recommended. The utility of microbiological sampling from macroscopically unremarkable tissue during TKA implantation remains controversial. Objective: To retrospectively evaluate the rate of periprosthetic joint infection (PJI) following TKA after PTOA and to assess the potential benefit of intraoperative microbiological sampling. The secondary objective was to evaluate the presence of prior colonization in osteosynthetic hardware among the affected cases. Patients and Methods: A retrospective screening of the hospital database was conducted between 2008 and 2022, including only AO/OTA type 41-B and 41-C fractures. Patients were assigned to a sampling group (with microbiological sampling during TKA) or a control group (without sampling). All patients received structured follow-up to assess postoperative complications. Results: A total of 40 patients met the screening criteria. In the sampling group (*n* = 29), 17.24% required surgical revision, and the rate of PJI was 3.45%. In the control group (*n* = 11), 18.14% underwent revision surgery, with a PJI rate of 9.09%. The average follow-up period was 4.35 years (range 2–11.6 years). Discussion: TKA in patients with PTOA is associated with a heightened risk of complications. A noteworthy possible correlation between systematic microbiological sampling and reduced PJI incidence was observed. While the small sample size limits definitive conclusions regarding causality, the findings support the potential value of consistent intraoperative sampling.

## 1. Introduction

Intra-articular fractures of the tibial plateau are severe injuries that affect the load-bearing surface of the knee joint. They are often associated with soft-tissue damage, including ligament and meniscal injuries, and may lead to joint incongruity, instability, and impaired mobility. Despite advances in surgical treatment, the management of these fractures remains challenging, particularly in cases with complex or comminuted fracture patterns. Various osteosynthesis techniques are available for the treatment of tibial plateau fractures, depending on the fracture type and soft-tissue condition. These include lateral or bilateral locking plate fixation, screw osteosynthesis for minimally displaced or depressed fragments, and hybrid constructs combining plates and screws. The goal of all these techniques is to restore joint congruity and provide stable fixation to allow early mobilization and reduce the risk of post-traumatic osteoarthritis (PTOA).

Intra-articular proximal tibial fractures cause PTOA with incidences between 23 and 44% [1]. Osteoarthritis itself is a complex pathology, which often results in interactions between biochemical inflammation processes and biomechanical causes [2]. PTOA is a form of osteoarthritis that develops as a result of a prior joint injury. It is characterized by progressive degeneration of articular cartilage, joint pain, stiffness, and functional impairment, and can occur months to years after the initial trauma. In most cases, total knee arthroplasty (TKA) is indicated to alleviate pain and preserve joint function. The procedure involves replacing the damaged or diseased articular surfaces of the femur and tibia with prosthetic components, thereby restoring mobility and improving quality of life. Patients show a 3.7-time greater risk for TKA implantation within the first three years after knee fracture [3]. Ten years after trauma, 7% of patients have an indication for TKA [4].

Performing TKA in patients with PTOA is challenging and implies increased complication rates such as superficial or deep infections, wound complications, patellar tendon rupture, or stiffness [5,6,7]. The reasons for this are various, e.g., poor soft-tissue envelope due to multiple prior surgeries, residual implants, multi-plane malalignment, or occult infection [7]. Total joint arthroplasty is typically performed as an elective procedure. As such, all modifiable risk factors are addressed preoperatively to optimize the patient’s condition and maximize the likelihood of a successful surgical outcome. Common modifiable risk factors include adequate glycemic control in patients with diabetes, weight reduction in cases of obesity, and smoking cessation. Implants from previous osteosynthesis at the tibial plateau often interfere with the correct positioning and alignment of knee prosthesis components. For technical reasons, hardware removal is therefore usually required prior to or during total knee arthroplasty to allow for proper implantation and to avoid malalignment or insufficient fixation of the tibial component. Implant removal and TKA can be done in one or two stages [8]. In their retrospective study with 4502 patients, Baker et al. found increased rates of periprosthetic joint infection (PJI) one year post-operatively when implant removal was performed concurrently or within three months before TKA [9]. Further complications include higher infection rates due to a compromised soft tissue, multiple prior surgeries, or occult infection [6].

A high bone-healing rate can be observed even under septic conditions; therefore, successful fracture consolidation should not be considered a reliable indicator of infection clearance [10]. High rates of implant colonization, with bacteria up to 55%, were found in patients undergoing elective implant removal [11]. Wrong negative microbiological findings are not unusual [12]. But the coincidental detection of pathogens does not appear to be a mandatory postoperative complication and is not equate to PJI [8]. Nonetheless, bacterial colonization of the implant should be ruled out before TKA but this remains challenging. Reversely, if pathogen detection is observed during implant removal, it remains unclear whether patients show a higher risk of PJI after TKA implantation.

TKA performed after knee injury or surgery carries a risk of specific complications, such as infection [13]. However, if the tissue does not show any macroscopic signs of infection, the operating surgeons are faced with the decision of whether to proceed with further microbiological examination. Unlike in oncological surgery, where intraoperative frozen section analysis enables just-in-time diagnostics, no equivalent exists for infection diagnostics. As a result, the surgical procedure must be completed despite diagnostic uncertainty remaining. However, performing diagnostic tests merely for formality without providing clinical benefit to the patient should be avoided, just as much as omitting a meaningful and necessary investigation that could influence the postoperative outcome. Therefore, the aims of this study were to evaluate the rate of PJI after TKA implantation with a minimum follow-up of two years and the possible benefit of microbiological sampling from macroscopically unremarkable tissue during TKA implantation. The secondary objective, in hindsight, was to evaluate the rate of prior asymptomatic colonization in osteosynthetic hardware among the affected cases and to compare it with rates reported in the literature. The results of this study aim to contribute to a better understanding of the clinical challenges and help clarify decision-making processes in this context. Due to the rarity of total knee arthroplasty following post-traumatic osteoarthritis, the study may also provide valuable baseline data for future meta-analyses.

## 2. Materials and Methods

### 2.1. Patients and Methods

The comprehensive hospital database was screened for patients undergoing TKA due to PTOA in the period from October 2008 to October 2022. During the screening process, patients meeting the following criteria were eligible for enrolment: PTOA after fracture types 41-B and 41-C of AO/OTA Trauma Classification compendium 2018 [14]. Type B fractures involve a partial articular surface, while type C fractures are more complex, involving the entire articular surface and metaphysis. All forms of internal osteosynthesis were accepted as eligible within the screening process. These covered the use of free screws, single and multiple plates, and the combination of free screws and plate osteosynthesis. All types of TKA following PTOA were considered during the screening process. This means posterior cruciate-retaining, posterior-constrained, and rotating hinge. Cruciate-retaining implants preserve the posterior cruciate ligament and rely on it for anteroposterior stability, offering more physiological knee kinematics. Posterior-constrained or posterior-stabilized designs replace the function of the posterior cruciate ligament with a cam-and-post mechanism, providing increased stability when the posterior cruciate ligament is absent or insufficient. Rotating hinge prostheses offer the highest degree of constraint through a mechanical linkage that allows flexion–extension and limited axial rotation. All TKA were inserted using cemented technique. As part of the screening process, patients who met the following criteria were deemed ineligible for enrolment: PTOA after fracture types 41-A; non-surgical treatment of the tibial fracture; unicompartmental knee replacement; additional femoral fractures; previous diagnosed chronic infections with contraindication to TKA; and a follow-up shorter than two years.

### 2.2. Methods of Assessment

The following parameters were collected retrospectively: gender, fracture type, number of surgeries until the fracture has been stabilized, duration between last osteosynthesis and TKA surgery as well as duration between implant removal and TKA surgery, age at TKA implantation, preoperative blood sample levels of c-reactive protein (CRP) and leukocytes, explanted osteosynthesis material. Moreover, microbiological samples and sonication at the time of implant removal were screened. Additionally, microbiological samples during TKA surgery were evaluated, if available. In all affected patients, 3–5 samples were taken during osteosynthesis removal at the interface between bone, soft tissue, and osteosynthesis. During TKA implantation, 3–5 samples were taken intra-articularly from the medial, lateral, and suprapatellar recessus. Intraoperative microbiological samples were collected in a standardized manner, consisting of both fluid components and tissue specimens from the surgical site. All samples were processed under sterile conditions. All samples were labeled. To enhance diagnostic sensitivity, cultures were incubated for 14 days, in accordance with current recommendations for low-grade infections and implant-associated conditions. PJI was defined according to the ICM criteria. The ICM criteria were introduced as a comprehensive scoring system integrating clinical, laboratory, microbiological, and histopathological parameters. In the absence of definitive diagnostic findings, the minor criteria provide a structured and nuanced approach [15]. In the case of positive samples, a distinction was made between contamination and infection after a detailed examination of the clinical course and the patient’s parameters. The establishment of an interdisciplinary consensus was supported by consultative input from the Department of Microbiology.

All surgical procedures were performed in accordance with standard operating procedures, with a single-shot prophylactic dose of cefazolin administered at the beginning of surgery. In cases of allergy, clindamycin was used as an alternative. In patients with previous surgeries, as in this study, antibiotic therapy was prolonged. If the microbiological results were negative, antibiotic treatment was discontinued. In cases with positive microbiological findings, targeted and pathogen-specific antibiotic therapy was continued for a duration of three months.

The database was screened for postoperative complications, including soft-tissue infection, PJI, or revision surgery after TKA for any other reason. The follow-up with a minimum of two years was performed on our outpatient clinic during routine follow-up controls.

### 2.3. Inclusion and Exclusion Criteria

Patients who underwent microbiological sampling during implantation of TKA were included in the sampling group; patients without sampling were excluded and served as the control group.

### 2.4. Statistical Analysis

The analysis was purely descriptive. Continuous variables are presented as mean, median, and standard deviation (minimum and maximum). Absolute numbers, proportions, and percentages are reported where appropriate and are described in detail within the accompanying text. No inferential statistical tests were performed. Data analysis was conducted using Microsoft Office Excel 2016 (Microsoft Corporation, Redmond, WA, USA).

### 2.5. Level of Evidence

Although descriptive in nature, this study benefits from a comparatively large sample size, providing robust and valuable insights into population-level trends and characteristics. Level of evidence IV (cohort study): the extensive dataset enhances the reliability and generalizability of the findings, making it a strong foundation for future analytical research.

### 2.6. Declaration of Generative AI and AI-Assisted Technologies in the Writing Process

During the preparation of this work, the authors used ChatGPT/OpenAI (Modeltype GPT-4), DeepL/DeepL SE (Version number 25.7.1.16851+ebcc14147793b12530a63064be019d8906503d25), and Microsoft 365 Copilot/Microsoft Corporation (Version 2506 Build 16.0.18925.20076) to improve the language and readability. After using this tool/service, the authors reviewed and edited the content as needed and take full responsibility for the content of the publication.

## 3. Results

A total of 40 patients were identified during the screening process. Comprehensive data were collected for all patients. After reviewing the available data, 29 patients met the inclusion criteria and were assigned to the sampling group. A total of 11 patients who met the exclusion criteria were assigned to the control group. The gender distribution was female *n* = 23 and male *n* = 17 (sampling group: female *n* = 16 male *n* = 13; control group: female *n* = 7 male *n* = 4). The age distribution at the time of TKA implantation was median 58, mean 59.48, range 36–84 (sampling group: median 58, mean 59.93, range 36–84; control group: median 58, mean 58.27, range 48–71), measured in years. Fracture characteristics were classified according to the AO/OTA classification. Furthermore, the number of fracture-related surgical procedures was evaluated, as well as the type of final osteosynthesis. Most fractures were classified as C3 fractures (total *n* = 22) and B3 fractures (*n* = 16). In most cases, a single surgical procedure was necessary (*n* = 26) followed by two surgeries (*n* = 11). Two plates were used in most of the cases (*n* = 17), followed by one plate (*n* = 11). The details are presented in Table 1. Prior to TKA implantation, laboratory findings in the screened cohort showed a leukocyte count with a median of 7.25 × 10^3^/µL and a mean of 6.95 × 10^3^/µL, range: 0.5–11.2 (sampling group: median 6.6, mean 6.57, range 0.5–11.2; control group: median 7.7, mean: 7.96, range 4.5–11). Furthermore, certain characteristic patterns were observed in CRP level, with a median of 2.5 mg/dL and a mean of 4.89 mg/dL range: <0.1–38.5 (sampling group: median 2.5, mean 5.34, range: <0.1–38.5; control group: median 0.9, mean 3.25, range: <0.1–12.4).

The interval between osteosynthetic fixation and planned hardware removal measured in years was median 1.45, mean 3.12, range 0.2–21.5 (sampling group: median 1.4, mean 3.19, range 0.2–21.5; control group: median 1.8, mean 2.93, range 1–7). The interval between hardware removal and TKA implantation measured in years was median 0.35, mean 3.7, range 0.1–28.4 (sampling group: median 0.3, mean 3.78, range 0.1–28.4; control group: median 0.8, mean 3.47, range 0.2–22.7). The interval between osteosynthetic fixation and TKA implantation measured in years was median 2.4, mean 6.8, range 0.1–33.7 (sampling group: median 2.2, mean 6.94, range 0.1–33.7; control group: median 3.5, mean 6.41, range 1.5–23.7). In most cases (*n* = 16), a posterior cruciate-retaining TKA was used. Some 14 patients received a posterior-constrained TKA and in 10 patients a rotating hinge TKA was necessary. Revision of the TKA was performed in five patients in the sampling group and in two patients in the control group, respectively. A PJI was found in one patient in each group. The median follow-up time was 3.9 years for the sampling group and 3.6 years for the control group. Detailed data on the TKA characteristics, following revisions, and follow-up are shown in Table 2.

During primary implantation of TKA, microbial colonization was detected in 3 out of 27 cases (10.34%). Among the isolates, *Micrococcus luteus* was detected in two cases and *Bacillus* sp. in one case. However, the detected colonization did not result in revision surgery during the follow-up period, neither as a result of PJI nor for any other cause. However, in one of the cases named above, abnormal findings were already observed during elective hardware removal. Intraoperative microbiological analysis revealed the presence of *Staphylococcus warneri*, *Stenotrophomonas maltophilia*, and *Staphylococcus capitis*. As a secondary objective, retrospective data were collected to investigate notable findings during the removal of osteosynthetic material.

In the sampling group (*n* = 29), microbiological samples were obtained during elective hardware removal in 22 patients. Among these, 9 cases showed previously asymptomatic bacterial colonization, corresponding to a rate of nine out of 22 cases (40.9%). The spectrum of detected bacterial species included the following (in descending order of frequency): *Staphylococcus epidermidis*, *Cutibacterium acnes*, *Staphylococcus hominis*, *Staphylococcus capitis*, *Staphylococcus saccharolyticus*, *Staphylococcus warneri*, *Staphylococcus aureus*, and *Stenotrophomonas maltophilia*.

In the control group (*n* = 11), microbiological samples were obtained during elective hardware removal in five patients. Among these, three cases showed asymptomatic bacterial colonization, corresponding to a rate of three out of five cases (60.0%). The detected bacterial species were *Staphylococcus capitis* and *Streptococcus sanguinis*.

In the overall screened cohort (*n* = 40), microbiological samples were obtained in 27 patients. Among these, 12 cases showed asymptomatic bacterial colonization during elective hardware removal, corresponding to a rate of 12 out of 27 cases (44.4%). For further details, see Table 3.

## 4. Discussion

The treatment of PTOA with TKA remains an exciting and challenging surgical endeavor. Less than 2% of patients with primary TKA have previously undergone osteosynthesis. We are therefore talking about a comparatively small number of patients overall [16]. As the rarity of a condition increases, so does the difficulty of studying its clinical course and treatment options. This poses challenges both for patients, who may face limited evidence-based care, and for physicians, who must make decisions under conditions of greater diagnostic and therapeutic uncertainty. The treatment of infections, especially PJI, requires substantial time and effort, both for patients and the healthcare system. Several studies consistently demonstrate that periprosthetic knee infections substantially increase healthcare expenditures and represent a major financial burden compared to routine arthroplasty procedures. The direct medical costs for operative treatment of an infected TKA are two-fold higher than the costs of similar aseptic revisions [17]. One of the most significant contributing factors was found to be the length of hospital stay, which accounted for 40% of the total cost and cost six times more in the revision cohort than in the uncomplicated TKA cohort [18]. This is precisely why new studies are an important part of gaining knowledge.

First, our findings—considered as incidental observations—demonstrate a high rate of peri-implant colonization during elective osteosynthesis removal and are consistent with the current literature. Knabl et al. determined the colonization rate of osteosynthesis implants in patients with no clinical or laboratory signs of infection, using two methods: conventional culture and polymerase chain reaction (PCR) of sonication fluid [11]. Thirty-two (56.1%) implants showed a positive result, either by culture or PCR, with coagulase-negative *staphylococci* being the most commonly identified microorganism (68.1%). Furthermore, the detection rate of the culture (50.9%) was significantly higher compared to PCR (21.1%). Fuchs et al. detected a high bacterial implant colonization rate of 27% regardless of the initial type of surgery using sonication cultures of implants. The most common pathogens were coagulase-negative *staphylococci* (46%) [8]. In our studies as well, *staphylococci* were the most represented species. *Staphylococci* tend to form biofilms on implant surfaces, which protect them from antibiotics and the immune system. They are commonly associated with both acute and chronic periprosthetic joint infections and can be difficult to treat and diagnose. Their tendency to form biofilms on implant surfaces plays a central role in their pathogenicity. Within a biofilm, bacteria are embedded in a self-produced extracellular matrix that adheres to the implant material. This biofilm acts as a physical and chemical barrier, significantly reducing the efficacy of antibiotics and shielding the bacteria from host immune responses. As a result, infections caused by *staphylococci* are often persistent, difficult to eradicate, and may present with non-specific or delayed symptoms [19]. There is a need to improve the reliable detection of both aggressive and slowly progressing infections. The use of sonication may further enhance the sensitivity of pathogen detection and is currently under discussion. Tani and his research group have discovered that sonication shows a greater sensitivity (77.04%) and specificity than conventional periprosthetic tissue cultures (55.73%). The specificities of the two methods were 98.11 and 94.34%, respectively [20]. These findings are congruent with the comparative literature [21,22,23].

However, a fundamental global problem with this topic of PJI is that different criteria are used worldwide to differentiate between PJI and contamination [12]. There is a lack of a single “gold standard” test, and the heterogeneity of study designs evaluating the accuracy of diagnostic modalities [24]. Despite the high contamination rates during osteosynthesis removal, the later total number of revision surgeries of TKA with proven PJI was only 5%. It can be deduced that colonization with bacteria in the context of osteosynthesis does not necessarily result in PJI. Bacterial colonization may occur without causing clinical symptoms or triggering an overt inflammatory response. This subclinical state can persist, especially in the presence of biofilm-forming organisms. Whether colonization progresses to manifest infection depends on multiple factors, including the bacterial load, virulence, host immune status, and the stability of the implant. As such, not all bacterial presence on implants during or after osteosynthesis should be interpreted as an active infection requiring extensive intervention. This distinction is critical to avoid overtreatment and to guide appropriate clinical decision-making [11]. However, there is a clear consensus in the literature that every manifest infection that has occurred increases the risk of further infections [25,26,27]. In the case of infection-related interventions, the risk of revision surgery within two years is more than doubled compared to the case of non-infection-related replacement surgery. Registry data show that during the first exchange surgery, significantly more procedures are non-infection-related than infection-related, while at third exchanges, the majority of procedures are septic [16]. These findings indicate that repeated surgical interventions are linked to an increased risk of PJI, and that the probability of diagnosing a PJI appears to rise with the number of previous procedures.

From the early beginning, everything possible should be done to prevent future PJIs. The high rate of pathogen detection leads the authors of this study to the conclusion that a two-stage operation with separate elective removal of the inserted osteosynthesis materials in TKA after PTOA is advisable. A noteworthy correlation between PJI and microbiological sampling at the time of the TKA implantation was observed: 3.45% of patients in the sampling group and 9.09% in the control group developed a PJI. Due to the small sample size, this finding indicates a correlation rather than a causal relationship. However, it underscores the importance of performing consistent and systematic microbiological sampling during implantation procedures.

Two major problems are contamination and unexpected positive cultures. Contamination can occur during osteosynthesis implantation, removal, or during TKA implantation and poses a challenge for the surgeon in all cases. It often leads to misdiagnosis, leading to unnecessary surgeries and use of antibiotics. Therefore, it is important to clearly differentiate between infection and contamination. A subtle and careful interpretation in those cases is crucial [28]. Additionally, a precise examination of clinical and histopathological results is mandatory [28]. Close cooperation between microbiologists, surgeons, and infectiologists is therefore advantageous, especially in borderline cases [29].

Several limitations must be acknowledged in this study. First, due to the rarity of the condition, even in a high-volume center, only a limited sample size can be expected. National data report a PJI rate of 0.2–2% following arthroplasty of major joints [30]. Moreover, less than 2% of patients undergoing primary TKA have a history of previous osteosynthesis. Consequently, the overall number of eligible patients is comparatively small, and the results presented may therefore reflect only a limited and potentially biased representation of the actual situation [16]. Furthermore, the retrospective study design carries an inherent risk of selection bias and incomplete data capture. Potential confounding factors, such as comorbidities unrelated to the affected joint, were not included in the analysis.

Ultimately, only clear treatment strategies with unambiguous algorithms are needed to guarantee the best possible treatment. Efforts are being made to improve guideline recommendations [24,31]. The insights of this study should contribute to the overall objective, to improve future meta-analysis and diagnostic prediction algorithms. The results presented can contribute to a better understanding of the problem of PJI in TKA after PTOA. Perhaps new research approaches like machine learning-based diagnostic system for prediction of PJI will improve diagnostic potency for surgical decision-making compared with the commonly used criteria [32,33]. Unlike conventional scoring systems, such as the ICM criteria, these models are capable of simultaneously analyzing large volumes of heterogeneous data, including laboratory markers, microbiological results, imaging findings, and patient-specific risk factors. This allows for a more comprehensive and individualized risk assessment. Moreover, machine learning algorithms can detect subtle patterns that may not be apparent to clinicians, enabling earlier and more accurate diagnoses, particularly in borderline or ambiguous cases. Additionally, these models can continuously improve as more data become available, making them adaptable and dynamic in contrast to static diagnostic criteria. However, until such models are validated and widely implemented in clinical practice, efforts to improve the diagnostic accuracy and management of PJI must continue through conventional means. This includes the refinement of existing criteria, standardized protocols for sample collection and analysis, interdisciplinary collaboration, and heightened clinical awareness. Optimizing established diagnostic pathways remains essential to ensure timely and effective treatment, especially given the complexity and potential severity of PJI.

## 5. Conclusions

Until large cohorts with unified datasets of high quality and comprehensive evaluation or meta-analyses are available, the authors recommend a separate elective removal of osteosynthesis materials including microbiological sampling in cases of PTOA with the need for TKA. According to actual consensus decisions and guidelines, intraoperative multiple microbiological samples during implantation of TKA might be helpful. Tissue sampling is advised even when no macroscopic signs of infection are present, if the patient has a history of joint surgery or prior intra-articular fractures.

## Figures and Tables

**Table 1:** Detailed data of fractures and osteosynthesis in number (n), proportion (n/N), and percent (%).

Type of Fracture	B1	B2	B3	C3
Sampling group	1/29 (3.45%)	0/29 (0%)	12/29 (41.38%)	16/29 (55.17%)
Control group	0/11 (0%)	1/11 (9.09%)	4/11 (36.36%)	6/11 (54.55%)
Total	1	1	16	22
**Number of procedures**	**1**	**2**	**3**	**4**
Sampling group	20/29 (68.97%)	7/29 (24.14%)	1/29 (3.45%)	1/29 (3.45%)
Control group	6/11 (54.55%)	4/11 (36.36%)	1/11 (9.09%)	0/11 (0%)
Total	26	11	2	1
**Type of osteosynthesis**	**Single plate**	**Screws only**	≥**two plates**	**Plates and free screws**
Sampling group	10/29 (34.48%)	1/29 (3.45%)	13/29 (44.83%)	5/29 (17.24%)
Control group	4/11 (36.36%)	0/11 (0%)	4/11 (36.36%)	3/11 (27.27%)
Total	14	1	17	8

**Table 2:** Detailed data on the TKA characteristics and following revisions in number (n), proportion (n/N) and percent (%), follow-up in years.

Type of TKA	Posterior Cruciate-Retaining	Posterior-Constrained	Rotating Hinge
Sampling group	8/29 (27.59%)	11/29 (37.93%)	10/29 (34.48%)
Control group	8/11 (72.73%)	3/11 (27.27%)	0/11 (0%)
Total	16	14	10
**Revision of TKA**	**Revisions in total**	**Of which with PJI**	**PJI**
Sampling group	5/29 (17.24%)	1/5 (20%)	1/29 (3.45%)
Control group	2/11 (18.14%)	1/2 (50%)	1/11 (9.09%)
Total	7/40 (17.50%)	2/7 (28.57%)	2/40 (5%)
**Follow-up**	**Time in years**		
Sampling group	median 3.9 mean 4.34 range 2.1–11.6		
Control group	median 3.6 mean 4.39 range 2–9.9		
Total	median 3.6 mean 4.35 range 2–11.6		

**Table 3:** Detailed data on the microbiological samples and the detected pathogens.

Microbiological Samples	TKA Implantation	Osteosynthesis Explantation
Sampling group	3/27 (10.34%)	9/22 (40.9%)
Control group	-	3/5 (60%)
Total	-	12/27 (44.4%)
**Detected pathogens**		
Sampling group	2× *Micrococcus luteus* 1× *Bacillus* sp.	5× *Staph. epidermidis* 2× *Staph. capitis* 2× *Cutibacterium acnes* 2× *Staph. hominis* 1× *Staph. aureus* 1× *Staph. saccharolyticus* 1× *Staph. warneri* 1× *Stenotrophomonas maltophilia*
Control group	-	2× *Staph. capitis* 1× *Strept. sanguinis*

## Data Availability

The raw data supporting the conclusions of this article will be made available by the authors on request.

## Abbreviations

The following abbreviations are used in this manuscript:

PJI	periprosthetic joint infection
PTOA	post-traumatic osteoarthritis
TKA	total knee arthroplasty
PCR	polymerase chain reaction
